# Transcription factors of the Nuclear Factor I (NFI) family control hepatocyte differentiation and cytochrome P450 activity in human liver

**DOI:** 10.1016/j.phrs.2025.107998

**Published:** 2025-11

**Authors:** Kathrin Klein, Oliver Burk, Roman Tremmel, Florian A. Buettner, Lea Kaestle, Werner Schroth, Thomas E. Muerdter, Diana Eccles, Anna-Christina Schmidt, Hanno Nieß, Ulrich M. Zanger, Matthias Schwab, Volker M. Lauschke

**Affiliations:** a Dr. Margarete Fischer-Bosch Institute of Clinical Pharmacology, Stuttgart, Germany; b University of Tuebingen, Tuebingen, Germany; c Faculty of Medicine, University of Southampton, Southampton, United Kingdom; d Department of General, Visceral and Transplantation Surgery, University Hospital LMU Munich, Munich, Germany; e HTCR-Services GmbH, Planegg, Martinsried, Germany; f University of Tuebingen, Departments of Clinical Pharmacology, and of Pharmacy and Biochemistry, Tuebingen, Germany; g Department of Physiology and Pharmacology and Center for Molecular Medicine, Karolinska Institutet and Karolinska University Hospital, Stockholm, Sweden; h Department of Pharmacy, The Second Xiangya Hospital, Central South University, Changsha, China

**Keywords:** Cytochrome P450, CYP2D6, Nuclear Factor I family, NFIB, Drug metabolism, Hepatocytes

## Abstract

Interindividual variability in hepatic drug metabolism and transport is well-known, and genetic variation in pharmacogenes impacts the efficacy and safety of drug treatments. Recent reports have indicated that the minor allele of the nuclear transcription factor I/B (*NFIB*), rs28379954 T > C, affects the metabolism of risperidone and clozapine, which are mediated by CYP2D6 and CYP1A2, respectively. First, we reanalyzed the association between rs28379954 T > C and CYP2D6 activity in three independent cohorts exposed to CYP2D6 substrates (propafenone, tamoxifen, and sparteine) which revealed no association. Next, to investigate the effects of all four NFIs expressed in human livers more broadly, 150 well-characterized hepatic tissue samples, along with data on expression quantitative trait loci (eQTL) and genome-wide association (GWA), were used. NFI expression levels significantly correlated with the mRNA and/or protein expression of multiple CYP genes (e.g. CYP1A1, CYP1A2, CYP2A6, CYP2C8, CYP2C19) which was confirmed for NFIA with the metabolism of CYP2C19, CYP1A2, and CYP2A6 probe substrates. While non-genetic factors (e.g. age, inflammation) also control NFI expression, genetic polymorphisms did not reach genome-wide significance. To validate the identified associations, siRNA-mediated knockdowns were used in primary human hepatocytes, followed by RNA sequencing and evaluation of differentially regulated pathways. We identified significant downregulation of several metabolic pathways related to hepatic functionality, PPAR signaling, and drug metabolism for NFIB, NFIC, and NFIX, whereas pathways associated with cancer biology were significantly induced. In summary our findings provide further insight into hepatic CYP regulation via the NFI network with implications for the understanding of interindividual variability of drug metabolism.

## Highlights

• Factors influencing nucIear factor I (NFI) expression or function appear to be largely non-genetic.
• NFI mRNA expression is significantly correlated with expression and/or function of CYP isozymes.
• NFI factors may act as positive regulators of enzymes for drug metabolism in human hepatocytes.
• Human NFI transcription factors play pronounced roles in the control of hepatic drug metabolism.

## Graphical Abstract

**Figure:**
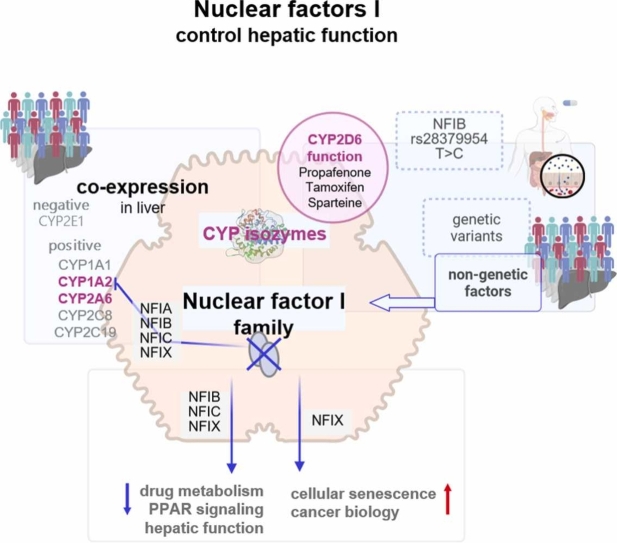

## 1 Introduction

Inter- and intraindividual variability of drug metabolizing enzymes (DME), transporters and their *cis*- and *trans*- acting regulators have been documented as major determinants of drug response [1], [2], [3]. Furthermore, age, sex, nicotine and alcohol consumption, nutrition, disease stages, as well as genetics, and drug-gene interactions are known individual factors contributing to variable expression and activity of cytochrome P450s but may explain only up to 40–50 % of variability [4], [5].

The Nuclear Factor I (NFI) family of transcription factors includes four coding (*NFIA*, *NFIB*, *NFIC* and *NFIX*) and two non-coding genes (*NFIA-AS1* and *NFIA-AS2*) in humans. NFI proteins contain SMAD/NF1 DNA-binding domains of the CCAAT-box binding type and play a broad role in developmental, physiological and pathological processes. Indicative of their importance is the finding that knockouts of *Nfia*, *Nfib* and *Nfix* are all embryonically or perinatally lethal in mice, while *Nfic* knockouts can survive to adulthood but show significant wound healing defects [6].

Over the past years, few reports indicated a role of NFI transcription factors in drug absorption, distribution, metabolism and excretion (ADME). Early reports postulated potential binding sites for NFIs in the promoter region of *CYP3A* genes that might impact isoform- and promoter-specific regulation of gene expression by interfering with YY1 binding [7], [8]. More recently, a role of NFIB in the regulation of CYP1A2 [9], [10], [11] and CYP2D6 [12], [13] was identified with potentially relevant clinical consequences for the metabolism of their respective substrates. Specifically, the minor allele of *NFIB* rs28379954T>C was reported to significantly increase CYP2D6-mediated risperidone metabolism about 2-fold in individuals classified as normal metabolizers (NM) based on *CYP2D6* genotype [12], suggesting that NFI factors may contribute to the interindividual variability in CYP expression. However, a comprehensive evaluation of the role of NFI family members on drug metabolism has not yet been conducted.

Here, we systematically analyzed the contribution of the the four coding and two non-coding human nuclear factor I genes on hepatic metabolism. After missing correlation of the *NFIB* rs28379954 T > C variant with CYP2D6 expression or function in three independent cohorts, effects of hepatic NFIs were more broadly investigated in a human liver bank with consequences on CYP-related drug metabolism. In addition, siRNA-mediated knockdowns were used in primary human hepatocytes (PHH), followed by RNA sequencing and evaluation of differentially regulated pathways to assess down- or upregulation of candidate genes with implications for the understanding of interindividual variability of drug metabolism.

## 2 Material and methods

### 2.1 Human liver samples

Liver tissue and corresponding blood samples for DNA extraction were collected from patients undergoing liver surgery at the Department of General, Visceral, and Transplantation Surgery at the Charité (Campus Virchow, University Medical Center Charité, Humboldt University Berlin, Germany) as previously described [14], [15]. A pathologist examined all tissue samples and only histologically non-tumorous liver tissue was collected and stored at −80 °C. In this study, we used 150 liver samples with detailed clinical documentation (Table 1; Supplementary Material Table S1**).** Genome-wide single nucleotide polymorphism data (Global Screening Array; Illumina*)* and gene expression data (HTA2; Affymetrix*)* were available for 150 and 118 samples, respectively [16], [17]. The expression of CYP450 proteins in liver microsomal preparations was immunoquantified (detailed in Supplementary Material Figure S1 and Supplementary Methods) or assessed by mass spectrometry [18], and the activities of CYPs were quantified using prototypical substrates, including propafenone, which is metabolized by CYP2D6, as previously described. [14], [15].

**Table 1:** Overview of study cohorts.

**Study**	**n**	**CYP2D6 drug substrate (activity measure) Ref**
TAM	579	Tamoxifen (MR=desmethyl-tamoxifen/endoxifen) [19]
SPART	199	Sparteine (MR_S_= sparteine/dehydrosparteine) [20], [21]
Liver bank	150	Propafenone; (microsomal propafenone 4-hydroxylation) [14], [15]

### 2.2 Human study cohorts

DNA samples and metabolic ratios (MRs) for the CYP2D6 substrates tamoxifen and sparteine, as well as their respective metabolites, were available from the TAM and SPART cohorts for CYP2D6 phenotype to genotype correlation analyses, as previously described [19], [20], [21] (Table 1).

In brief, to analyze the effect of genetic variants in *NFIB* gene on CYP2D6 dependent metabolism of tamoxifen in the TAM cohort we analyzed the 279 postmenopausal (German cohort) and 308 premenopausal (UK cohort) breast cancer patients treated with 20 mg/day adjuvant tamoxifen [19]. Of the 587 cases, germline DNA and plasma samples were available for 579 cases. Plasma samples were collected in a steady-state to estimate the concentrations of the CYP2D6-related tamoxifen metabolites, N-desmethyl tamoxifen and endoxifen. Key polymorphisms of functional *CYP2D6* alleles were genotyped by MALDI-TOF and genotype based activity scores were deduced [22]. The TAM Cohort was described in detail previously [19]. In addition, we used DNA samples from 199 healthy German individuals from the SPART cohort who were phenotyped with the CYP2D6 probe drug sparteine by determining the sparteine to dehydrosparteine MR, as previously described [20], [21]. Information on genetic variants of *CYP2D6*, as well as copy number variations (i.e. deletions, duplications), was available, enabling the assignment of CYP2D6 activity scores and a comprehensive genotype/phenotype association analysis. Details for the SPART study have been previously described [20], [21]. Finally, the aforementioned human liver bank was used as additional, independent cohort to evaluate the effect of the *NFIB* variant on CYP2D6-dependent propafenone hydroxylation. For this, CYP2D6 activity quantified in microsomes was cross-referenced with microsomal, immunoquantified, or mass spectrometry-based CYP2D6 protein expression, as previously described [14], [18]. Together with *CYP2D6* genetic information including the most common functional haplotypes, copy number variants, and genotype-derived activity scores, detailed genotype/phenotype association analyses were performed.

### 2.3 Genotyping of NFIB rs28379954

Genomic DNA samples of the TAM-cohort were genotyped by MALDI-TOF mass spectrometry-based genotyping (MassArray, Agena) using iPLEX chemistry with PCR primers forward (5′-acgttggatgTAAGGCCATACTGTCTCATC-3′), reverse (5′-acgttggatgCTTTGGTTCATGGTTTATTT-3′) and extension primer (5′-TTCTTAATTTCTAGGTAGTTGAG(A/G)-3′) at standard conditions. The genomic DNA samples from the SPART-cohort and the liver biobank samples were genotyped using a predesigned genotyping assay mix (C_59359617_10; Thermo Fisher) and allelic discrimination analysis was performed on a 7900 HT Fast Real-Time PCR-System (Applied Biosystems) according to the manufacturer’s recommendations. Genotyping results were in accordance with Hardy-Weinberg equilibrium (all Chi^2^ values were <5; Supplementary Material Table S2).

### 2.4 Primary human hepatocytes

Primary hepatocytes (PHH) were commercially acquired from BioIVT (UK) or isolated from liver tissue samples of patients, who underwent partial hepatectomy at the Department of General, Visceral, and Transplantation Surgery of the Ludwig-Maximilians University Munich using a two-step collagenase perfusion technique [23], [24]. Tissue samples and annotated data were obtained within the framework of the non-profit foundation Human Tissue and Cell Research, HTCR [25]. Donor data are shown in Supplementary Material Table S3.

### 2.5 Knockdown experiments

Isolated primary human hepatocytes were seeded at 1.0 × 10^6^ cells per well in William’s E medium supplemented with 10 % defined fetal bovine serum (FBS Gold, PAA laboratories, Cölbe, Germany), 2 mM L-glutamine, 100 U/ml penicillin, 100 µg/ml streptomycin, 0.1 % DMSO, 0.03 IU/ml insulin and 100 nM dexamethasone into collagen type I-coated 6-well plates. After 10–12 h, the medium was replaced by fresh medium without antibiotics and cells were transfected with 20 nM siRNA using Lipofectamine RNAiMAX (Life Technologies). Gene-specific Silencer Select siRNAs (Life Technologies) targeting NFIA (s9476), NFIB (s9494), NFIC (s9497) and NFIX (s9503) and non-targeting negative control siRNA (Silencer Select negative control 1) were used. Culture medium was renewed 24 h and 48 h after the start of transfection. After 72 h, total RNA was isolated and knockdown efficiencies were determined by RT-qPCR or RNAseq.

### 2.6 Transcript analyses

Total RNA was isolated using the NucleoSpin RNA kit (Macherey-Nagel, Düren, Germany), including on-column digest of residual DNA by DNaseI. RNA integrity was determined with the RNA 6000 Nano Kit, using a 2100 Bioanalyzer System (Agilent) and samples with high RNA integrity (RIN>9.0) were further processed. TaqMan qPCR analyses were conducted using the primers and probe previously described for CYP2B6 [26] or using commercial gene expression assays (Life Technologies), consisting of pre-designed primer/probe sets (Supplementary Material Table S4). Assays were run in technical triplicates on a Biomark HD system with FlexSix gene Expression Integrated Fluidic circuits (Standard Biotools, South San Francisco, CA), as described previously [27] and gene expression levels in samples were calculated relative to TBP levels using the ΔΔCt method.

Knockdown experiments for each *NFI* gene were run in triplicates. RNA-sequencing was performed at GenomeScan BV (Leiden, The Netherlands) by poly-A capture using 150 bp paired-end reads and 20 million reads per sample. Raw RNA sequencing data were processed using the nf-core/rnaseq pipeline (v3.14.0), which included UMI processing, alignment to the GRCh38 genome, and expression quantification with Salmon [28], [29]. Salmon was run in alignment-based mode with the --seqBias and --gcBias options enabled. Counts were quantified based on Ensembl gene annotation (v111). For differential expression analysis, we used DESeq2 (v1.36.0). Significant changes in gene expression were identified using a fold change threshold of 1.5 and an FDR-adjusted p-value of p_adj_< 0.05. Gene set enrichment analyses were performed using the WEBGestalt toolbox [30] on lists with differentially expressed (DE) genes p < 0.05, preranked by log2(fold change) values < -1 or > 1 compared to the respective controls. Redundancy reduction was applied to correct for overlapping gene sets and significance was set at FDR< 0.05. Leading edge subset of genes refers to the core group of genes in a set contributing most to the enrichment signal. Expression changes of *CYP* genes under NFI depletion was further validated in four additional independent hepatocyte donors (Supplementary Material Table S3**)** and quantified as described above using Biomark platform and assays as listed in Supplementary Material Table S4.

### 2.7 CYP activity profiling

CYP enzyme activities in PHH cultures were determined using a LC-MS/MS substrate cocktail assay 72 h after siRNA transfection. Hepatocytes were incubated with a cocktail of CYP substrates in culture medium without DMSO, consisting of 50 µM phenacetin (CYP1A2), 25 µM bupropion (CYP2B6), 5 µM amodiaquine (CYP2C8), 35 µM atorvastatin (CYP3A4), 5 µM propafenone (CYP2D6), 100 µM tolbutamide (CYP2C9), and 100 µM *S*-mephenytoin (CYP2C19). After 3 h or 4 h, an aliquot of the incubation medium was adjusted to final 50 mM of formic acid and deuterium labeled internal standards for each metabolite, and metabolites were quantified as described [31], [32].

### 2.8 Statistical analysis

Correlation analyses of NFI gene expression data with CYP mRNA, protein, and activity endpoints retrieved from the liver cohort were performed using non-parametric Spearman correlation. Associations with Benjamini-Hochberg (BH) corrected p_*adj*_ < 0.05 was considered as significant. Statistical analyses were conducted in GraphPad Prism v10 (GraphPad, CA, USA), SRplot [33] and R studio. Venn diagrams were generated using Venny2.1 (https://bioinfogp.cnb.csic.es/tools/venny/). Some illustrations were generated with BioRender.com.

## 3 Results

**Fig. 1:**
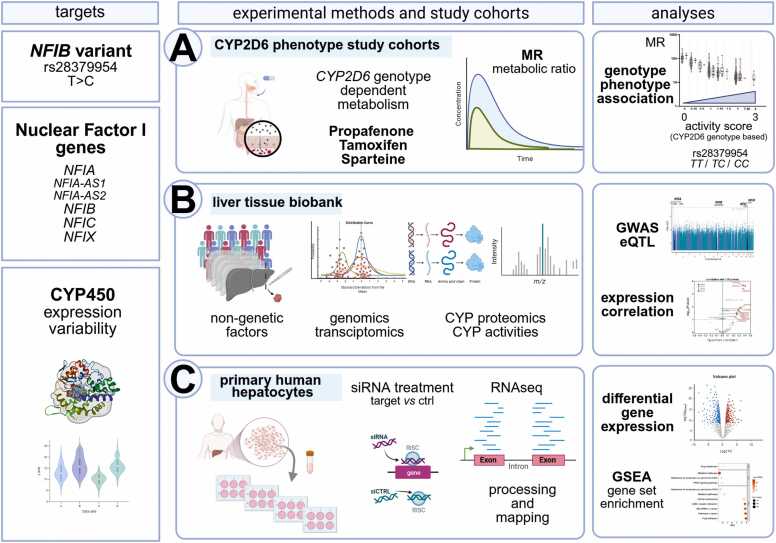
Experimental strategy to investigate the role of NFI transcription factors in cytochrome P450 gene expression. Three complementary strategies were applied. A: Association of the *NFIB* rs28379954 variant with different genotype-based CYP2D6 activity scores for the CYP2D6 substrates propafenone, tamoxifen, and sparteine, and their respective metabolic ratios (MR), in three independent human cohorts. B: Correlation analyses of the mRNA expression of NFI family members with non-genetic factors, CYP450 enzymes and genome-wide factors in the human liver cohort (n = 118). C: Treatment of primary human hepatocytes with siRNAs targeting NFIA, NFIB, NFIC, as well as a non-targeting control and subsequent transcriptomic profiling using RNA sequencing, followed by differential gene expression analysis. [Created with BioRender.com].

To evaluate the regulatory landscape of NFI transcription factors, we adopted both a targeted approach as well as a comprehensive profiling angle. First, we aimed to reanalyze the previously reported association between *NFIB* rs28379954T>C and CYP2D6 activity in independent human cohorts exposed to different drugs (Fig. 1A). Subsequently, we systematically investigated associations of the expression of NFI family members with non-genetic factors, the expression and function of CYP450 enzymes and on a genome-wide level in the IKP liver tissue biobank (Fig. 1B). These data were complemented by knockdown experiments in PHH coupled to RNA-sequencing and functional profiling to parse the network of NFI target genes in human liver cells (Fig. 1C).

### 3.1 The well-characterized candidate variant *NFIB* rs28379954T>C is not associated with CYP activity in three independent cohorts

**Fig. 2:**
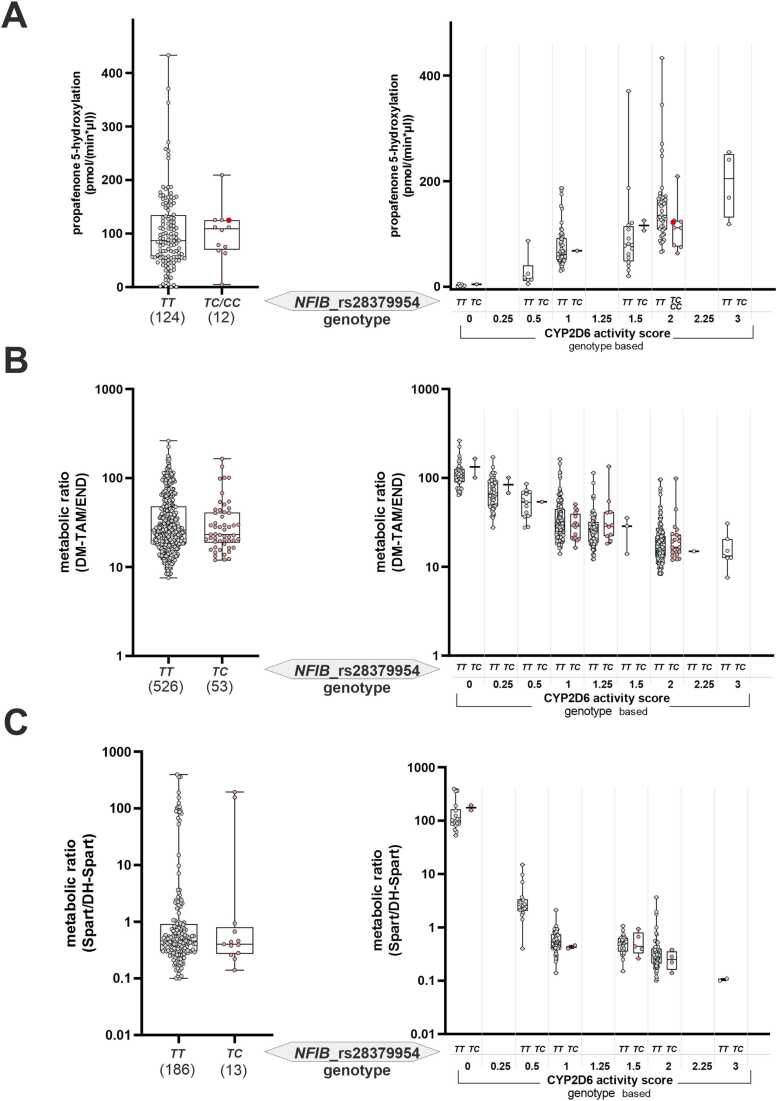
Assessment of the impact of the *NFIB* rs28379954T>C variant on CYP2D6 mediated drug metabolism. A: CYP2D6 propafenone-5-hydroxylation activity (pmol/(min*µg)) measured in human liver microsomes (n = 138). B: Metabolic ratio of desmethyl tamoxifen (DM-TAM) to endoxifen (END) levels in the TAM study cohort (n = 579). C: Metabolic ratio of sparteine (Spart) to dihydrosparteine (DH-Spart) in the SPART cohort (n = 199). *NFIB* rs28379954 TT carriers (grey circles) are comparted to TC/CC carriers (red circles); all individual data points are shown, whiskers ranging from minimal to maximal values, boxes displaying the interquartile range (25th and 75th) and median.

The variant rs28379954 had been identified in clozapine-treated patients based on its association with reduced clozapine plasma levels [11] and subsequent work suggested a pronounced effect of this variant on the CYP2D6-dependent metabolism of risperidone [12]. To investigate the substrate-specificity of these reports, we evaluated associations between rs28379954C-carrier status and CYP2D6 activity in three independent cohorts (TAM cohort, SPART cohort, IKP liver bank) using diverse CYP2D6 substrates. Notably, we did not find differences in CYP2D6 activity based on *NFI* genotype in any of these cohorts. Specifically, rs28379954 was not associated with propafenone-5-hydroxylation activity quantified in microsomes from 138 samples of the IKP liver biobank (Fig. 2A). Neither did we observe significant associations with the desmethyl-tamoxifen/endoxifen MRs in the TAM cohort (579 pre- and postmenopausal breast cancer patients) (Fig. 2B) nor with sparteine/dehydrosparteine MRs in the SPART cohort of 199 healthy German volunteers (Fig. 2C). These results indicate that the effect of NFI transcription factors on hepatic CYP metabolism might be substrate- or cohort-specific.

### 3.2 NFI transcription factor expression correlates with CYP expression and drug metabolism

**Fig. 3:**
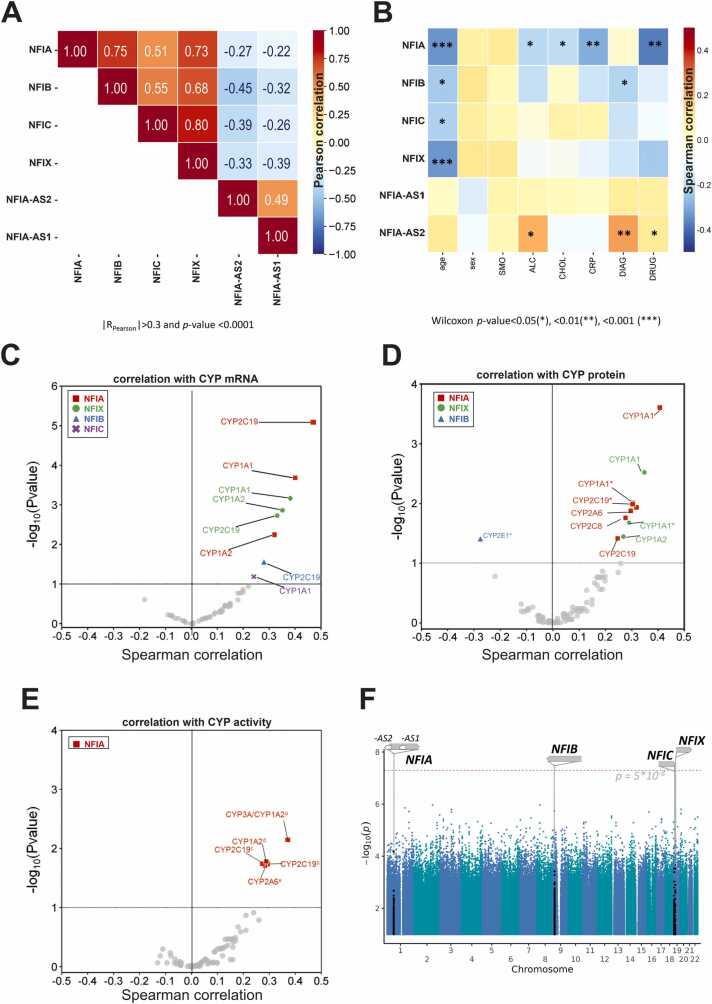
Correlation of NFI expression with genetic and non-genetic factors in a human liver cohort (n = 118). A: Pearson correlation coefficients (R_Pearson_) between NFI members to each other (blue, negative correlation; red, positive correlation; values given for R_Pearson_>0.3 and p < 0.0001). B: Impact of non-genetic factors: sex (female n = 63 *vs* male n = 55), smoking (SMO n = 95 *vs* non-SMO n = 21); alcohol consumption (ALC n = 38 *vs* no ALC n = 77); cholestasis (CHOL, no n = 21 *vs* yes n = 93); C reactive protein (CRP elevated levels n = 6 *vs* normal levels n = 109); underling liver disease (DIAG, patients with isolated liver metastasis n = 76 *vs* patients with benign or malign primary liver tumors n = 41) and co-medication (DRUG n = 32 vs no medication n = 31). R_Pearson_ correlation and Spearman correlation coefficients are given for age and all other factors, resp. Wilcoxon p-values marked as p < 0.05(*); p < 0.01(**); p < 0.001(***). C-E: Spearman correlations with regard to selected CYP450 enzymes: mRNA (C); protein (D) quantified by Western Blot or mass spectrometry (marked with *); microsomal CYP450 activity (E) determined with prototypical substrates: ^a^ propafenone-N-desalkylation (CYP1A2/CYP3A); ^b^ S-mephenytoin-4-hydroxylation (CYP2C19); ^c^ R-omeprazole-5-hydroxylation (CYP2C19); ^d^ phenacetin-O-deethylation (CYP1A2); ^e^ coumarin-4-hydroxylation (CYP2A6). Only associations with Benjamini-Hochberg corrected p_adj_< 0.05 are shown. Symbols: square/red, correlation with NFIA expression; triangle/blue, correlation with NFIB expression; cross/pink: correlation with NFIC expression; dot/green, correlation with NFIX. F: Manhattan plot of eQTL analysis of mRNA expression for the four coding NFI genes; chromosomal gene regions and the adjusted p-value level for significance at 5*10^−7^ are marked by arrows and dashed lines, respectively.

Next, we evaluated the effects of hepatic NFI transcription factors more comprehensively. To this end, we first evaluated their relative expression levels in 118 human liver samples using HTA2 global gene expression data. The expression variability of both the coding and non-coding NFI genes *NFIA*, *NFIB*, *NFIC*, *NFIX*, *NFIA-AS1* and *NFIA-AS2* was normally distributed with moderate coefficients of variation (2.6 % up to 5.3 %). Interestingly, expression of the four coding genes was positively correlated (ρ>0.5), particularly for *NFIA* and *NFIB* (ρ=0.75) and *NFIC* and *NFIX* (ρ=0.8; Fig. 3A). In contrast, both non-coding genes *NFIA-AS1* and *NFIA-AS2* were in anti-correlation with all four *NFI* genes and most pronounced with *NFIB* (ρ=-0.45; Fig. 3A), suggesting inhibitory roles of these transcripts. Age was in significant negative correlation with the expression levels of all *NFI* genes (Fig. 3B, Supplementary Material Figure S2A). Furthermore, in group comparisons expression of *NFIA* but not of the other *NFI* genes was lower with alcohol use, elevated levels of CRP (inflammation), cholestasis and the use of CYP inducers (Fig. 3B, Supplementary Material Figures S2B-E).

Notably, NFI expression levels correlated significantly with the mRNA expression of multiple *CYP* genes, including CYP1A1 (NFIA, NFIC, and NFIX), CYP1A2 (NFIA and NFIX) and CYP2C19 (NFIA, NFIB and NFIX; Fig. 3C). At the protein level, correlations between CYP enzyme expression and NFI expression were confirmed for CYP1A1 (NFIA, NFIX), CYP1A2 (NFIA, NFIX) and CYP2C19 (NFIA). Additional correlations were identified for CYP2A6 (NFIA) and CYP2C8 (NFIA), as well as a single negative correlation for CYP2E1 (NFIB; Fig. 3D).

Particularly for NFIA, these associations were further confirmed at the functional level by significant positive correlations with the metabolism of various probe substrate activities for CYP2C19 (*S*-mephenytoin-4-hydroxylation, *R*-omeprazole-5-hydroxylation), CYP1A2 (propafenone-*N*-desalkylation, phenacetin-*O*-deethylation) and CYP2A6 (coumarin-*4*-hydroxylation) (Fig. 3E). However, a genome-wide association study (GWAS) of genetic factors that might explain some of the observed variability in NFI expression did not reveal any single nucleotide polymorphisms that reached genome-wide significance (p < 5*10^−8^; Fig. 3F). This corroborates and further extends the observations described above for rs28379954. Taken together, these results indicate that human NFI transcription factors play pronounced roles in the control of hepatic CYP expression. However, the factors influencing NFI activity appear to be primarily non-genetic.

### 3.3 NFI transcription factors are involved in regulating the expression of drug metabolizing enzymes in human liver

**Fig. 4:**
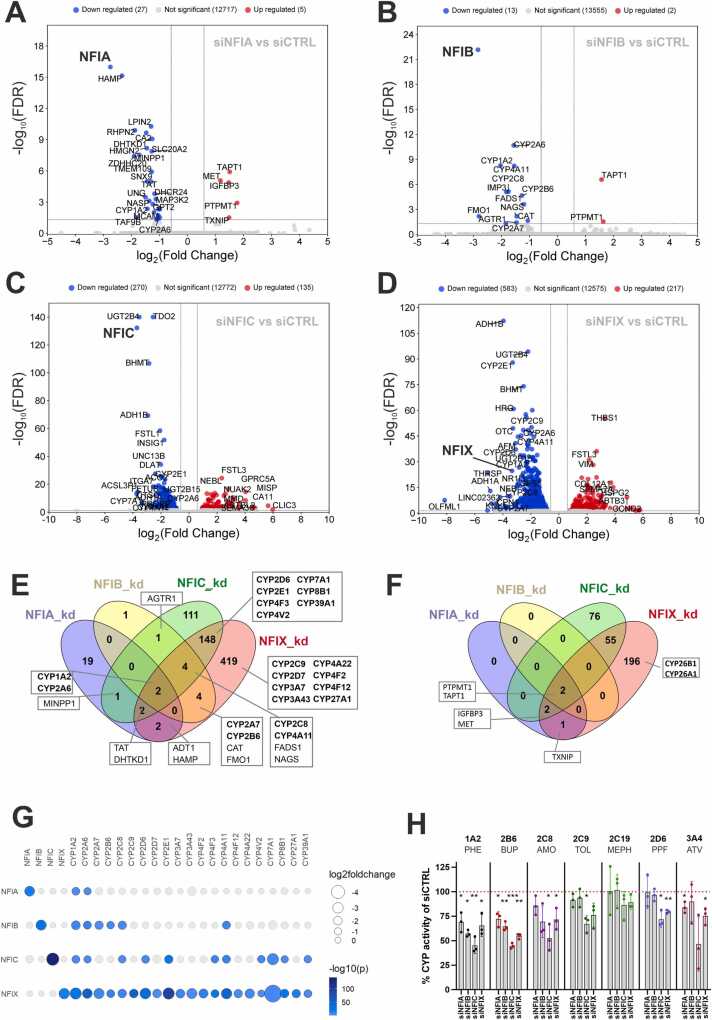
Knockdown experiments of NFIs in primary human hepatocytes and its consequence for CYP450 enzyme expression. A-D: Downregulated and upregulated genes for each knockdown after 72 h shown as log2(fold changes) compared to siCTRL treated cells; blue, lower expressed; red, higher expressed *(*p_adj_<0.05 and FC>1.5 or FC<-1.5). FC, fold change. E-F: Venn diagram for the comparison of significantly downregulated (E) and upregulated (F) genes (p_adj_<0.05); CYP450 isozymes in subsets are named in bold. G: Comparison of expression differences for several *CYP450* genes upon knockdown of *NFI* factors. *NFI* and *CYP450* genes are shown with at least one significant in knockdown setting; circle size: log2(fold changes); color code: -log10(*p*_adj_). H: Changes of CYP450 activities upon *NFI* gene knockdown in independent PHH donors. Abbreviations: PHE, phenacetin O-deethylation (CYP1A2); BUP, bupropion 8-hydroxylation (CYP2B6); AMO, amodiaquin N-desethylation (CYP2C8); ATV, atorvastatin o-hydroxylation (CYP3A4); PPF, propafenone-5-hydroxylation (CYP2D6); TOL, tolbutamide 4-hydroxylation (CYP2C9); MEPH, S-mephenytoin 4-hydroxylation (CYP2C19); Boxes, mean of three biological replicates; whiskers, standard deviation. Statistical significance was tested between siNFI treatment versus siCTRL treatment using one sample *t*-tests, significant p-values are given as p < 0.05(*); p < 0.01(**); p < 0.001(***).

To provide functional support for the observed associations, we used siRNA-mediated knockdowns in PHH for the coding NFIs (NFIA, NFIB, NFIC, NFIX) followed by RNA-sequencing**.** All knockdowns significantly and specifically reduced the expression levels of the respective target gene, without strongly affecting the expression of other NFI family members (Supplementary Material Figure S3A). However, some cross-reactivity could be observed for siNFIC, which resulted in a 60–80 % reduction of NFIA expression in all donors and may partially contribute to the differentially expressed genes observed upon NFIC knockdown. Knockdown of NFIX (n = 678) and NFIC (n = 328) resulted in the largest numbers of (DE) genes, whereas siRNAs against NFIA and NFIB only impacted the expression of 28 and 13 genes, respectively (Fig. 4A-D). Only four genes were shared among all *NFI* knockdowns. These included *CYP1A2* and *CYP2A6*, which were ubiquitously downregulated (indicated in Fig. 4E) and *PTPMT1* and *TAPT1*, which were consistently upregulated (as shown in Fig. 4F). Notably, it appears these genes are not impacted by transfections *per se* since they did not overlap with impacted genes identified in previous knockdown studies of *CYP* genes in human hepatocytes [34]. Multiple other *CYP* genes were also differentially expressed upon knockdown of *NFIB*, *NFIC* and *NFIX* (Fig. 4G). Impaired *CYP* gene expression was confirmed by *NFI* gene knockdown in four additional hepatocyte donors (Supplementary Material Figure S3B) and the functional effects were moreover validated in three of these donors by profiling the activities of seven CYPs using a probe cocktail approach (Fig. 4H). CYP1A2 and CYP2B6 metabolism was consistently reduced across all *NFI* knockdowns, while the activities of CYP2C8, CYP2C9, CYP2D6 and CYP3A4 were only affected by knockdown of *NFIX* and/or *NFIC*. The results thus confirm that downregulation of *NFI* genes reduces CYP-mediated drug metabolism and particularly reduced activity of NFIX and NFIC causes broad reduction in DME activities.

**Fig. 5:**
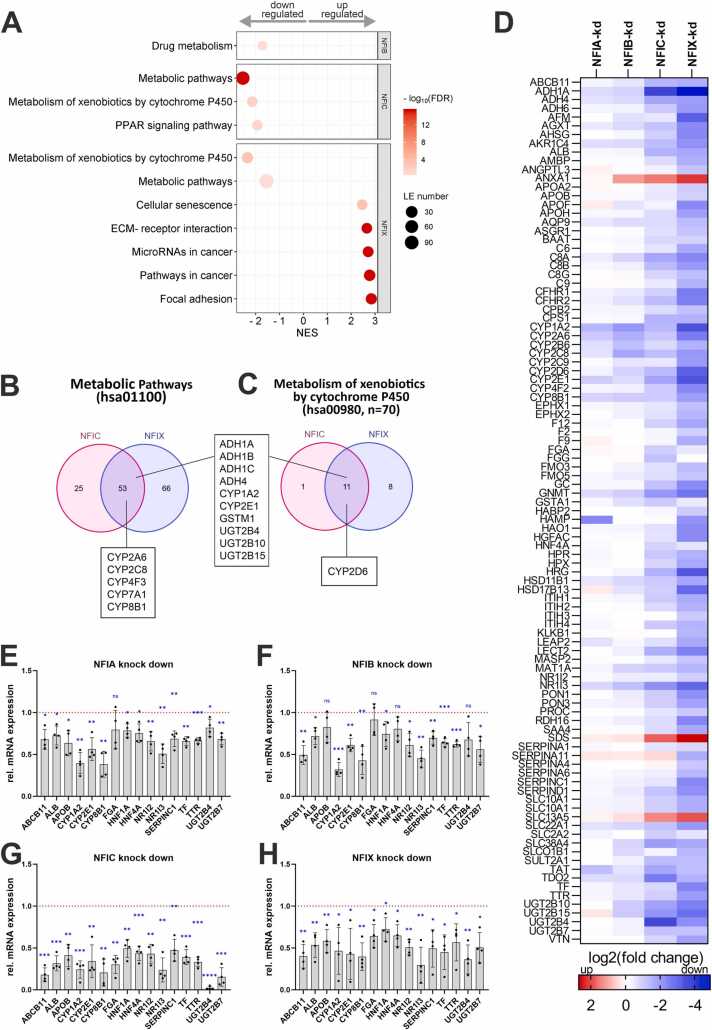
Pathway analyses of differentially expressed genes in primary human hepatocytes with *NFI* knockdown. A: KEGG pathway terms for up- and downregulated genes sorted according to normalized enrichment score (NES, x-axis); color-scale: -log10(FDR p-value); dot size: number of core genes in leading edge (LE) core genes. B-C: Venn diagrams of enriched genes in the KEGG pathways “Metabolism of xenobiotics by cytochrome P450” (hsa00980) and “Metabolic pathways’ (hsa01100) for NFIC and NFIX knockdowns. Drug metabolizing enzymes are shown for the overlap of the single KEGG pathway terms, as well as between them. D: Expression levels in siRNA treated hepatocytes compared to siCTRL treatment derived from RNAseq data shown as log2(fold changes) and color coded for each knockdown. From a set of 126 marker genes, 99 genes with at least one significant expression change upon NFI knockdown are shown. E-H: Selected hepatocyte marker genes were re-analyzed using Taqman assays on a Fluidigm system in four independent donors and displayed as relative mRNA expression compared to siCTRL treatment. One sample *t*-tests were performed; p ≤ 0.05(*); p < 0.01(**); p < 0.001(***); p < 0.0001(****). ns = not significant (p > 0.05).

To assess the biological effects of *NFI* genes in human hepatocytes beyond drug metabolism, we evaluated the differentially regulated pathways. No changes at the pathway level were detected for NFIA, likely due to the low number of significantly affected genes (n = 28). For NFIB, NFIC and NFIX, we could identify significant downregulation of several metabolic pathways related to hepatic functionality, such as metabolism of xenobiotics, PPAR signaling, and drug metabolism (Fig. 5A). When analyzing the leading edge core genes of the differentially affected gene set ‘Metabolic pathways’ (hsa01100) in more detail, we found that besides several DMEs, *CYP1A2*, *CYP2A6*, *CYP2C8*, and *CYP2E1* were shared between NFIC and NFIX downregulated genes (Fig. 5B). Furthermore, the gene set ‘Metabolism of xenobiotics by cytochrome P450’ (hsa009890) was found to be differentially regulated in both NFIX and NFIC knockdown conditions, and the overlap of core genes covers 11 genes, including CYP1A2, CYP2D6, CYP2E1, as well as several members of the *ADH* and *UGT2B* gene families (Fig. 5C). Importantly, the expression of differentiated hepatocyte markers was significantly reduced (25 % up to >90 %) upon knockdown of NFI factors more broadly (Fig. 5D-H). This included the majority of canonical hepatocyte markers from the literature [35], [36], [37], [38], including albumin, lipoproteins (APOB), nuclear hormone receptors (PXR (NR1I2) and CAR (NR1I3)), transferrin, transthyretin as well as the key hepatic differentiation factors HNF4A and HNF1A. In contrast, genes that are considered to be elevated in fetal or dedifferentiated hepatocytes, such as ANXA1 (FC=4.4) and GSTP1 (FC=1.4) tend to be upregulated upon NFI knock-down. Combined, these results suggest that NFI transcription factors contribute towards the maintenance of a mature hepatocellular phenotype, whereas their loss entails hepatic dedifferentiation, which may pave the way for cellular transformation.

## 4 Discussion

Interindividual differences in drug metabolism are caused by both genetic and non-genetic factors. Pharmacogenetic twin studies that compared mono- and dizygotic twins found large differences in the importance of genetic variation on drug metabolism with heritable factors explaining between 34 % and 98 % of the variability between participants [39]. However, common candidate variants only explain less than half of this genetic contribution [40]. Multiple hypotheses have been suggested to explain the missing heritability of pharmacogenetic traits, including rare variations that are not commonly tested or epistatic effects that arise from the interaction between different variations in *cis* or *trans* [41], [42]. Our data suggest that NFI transcription factors might play an important role in the regulation of hepatic drug metabolism. However, we do not find evidence for a strong genetic component of NFI variants. As such, our work supports results from a previous cocktail study in 200 healthy volunteers that also failed to identify a significant association between rs28379954 in *NFIB* and CYP2D6 mediated metabolism [43]. Instead, we identified different non-genetic factors that were associated with NFI expression, including inverse correlations with age and different factors of hepatocyte stress, such as alcohol intake, inflammation, and cholestasis. A limitation to the generalizability of the study is that it did not consider gender/sex issues. We conclude that transcription factors of the NFI family appear to be prominent modulators of hepatic CYP activity, including but not limited to CYP2D6; however, whether and to what extent CYP activity is impacted by genetic variation in *NFI* genes is a subject of debate and requires further investigations.

Knock-down of NFI transcription factors consistently down-regulated CYP gene expression, thereby indicating a respective activating role of NFIs. In accordance, previous work has demonstrated the existence and functional relevance of NFI binding motifs in the promoter regions of several mammalian CYP genes, including human CYP1A2 and CYP3A4/7, by gel shift assays, DNaseI footprinting and reporter gene analyses [8], [44], [45]. These studies suggest that NFIs activate the respective CYP genes by direct binding to motifs in their regulatory regions. However, definite proof for this assumption and generalization to further CYP and DME genes, identified here as being regulated by NFIs, require establishing the cistromes of NFIs in human hepatocytes combined with massively parallel reporter assays.

Chromatin immunoprecipitation of the different NFIs indicated considerable similarities in binding motifs [46]. Furthermore, *in vitro* profiling of NFI interactomes in neuroblastoma cell lines revealed similarities between the different NFI family members, which included interactions with chromatin remodeling and transcription regulation complexes, including the SWI/SNF and Mediator complexes [46]. Combined, these data suggest co-regulation of these genes and a potential overlap of transcriptional targets [47]. Consistent with these reports, we find that RNA expression of the different *NFI* genes was highly correlated in biopsies from 118 human livers. Furthermore, we observed that genetic interference using siRNA transfections resulted in partially overlapping affected gene sets despite high knockdown specificity, again indicative of considerable downstream redundancy. However, less is clear about the factors regulating NFI expression and activity. The high number of transcription factor interactions found with NFIs suggests considerable cross-regulation between NFI activity and other transcription factors [48]. Furthermore, we cannot exclude functional modulation via alternative splicing, which has been reported for most NFIs [49], [50]. These events will be interesting avenues for further investigations into the role of NFIs in human liver going forward.

Most research on NFI transcription factors has focused on their roles in musculoskeletal and neural development and stem cell maintenance [51], [52], [53], [54]. In contrast, in adult tissues they appear to rather promote the quiescence of adult stem cells. These lines of evidence have been generated in diverse contexts, including adult brain, bone marrow and hair follicle cells [55], [56], [57]; however, to the best of our knowledge, no studies have investigated effects of NFI on differentiation and stemness in adult liver. The adult liver is peculiar in its regenerative response – instead of having a dedicated stem cell pool, hepatocytes themselves can be considered as facultative stem cells. Under homeostatic conditions, hepatocytes are extremely long-lived with hepatocyte lifespans of years to decades [58]. However, upon liver injury, hepatocytes can rapidly re-enter the cell cycle and undergo compensatory hyperplasia resulting in the regeneration of up to 1 kg of liver tissue in less than six weeks [59]. This proliferative capacity is almost unlimited, as demonstrated by serial transplantations in which grafted hepatocytes could repopulate the injured liver parenchyma for at least 12 consecutive rounds [60]. The results presented here provide evidence that NFI transcription factors act in primary human hepatocytes in a way that is similar to their role in conventional adult stem cells in other tissues, i.e. by maintaining differentiation and contributing to the maintenance of cellular quiescence. For a hepatocyte to enter this regeneration program, it must dedifferentiate and inhibition of TGFβ signaling is required for this quiescent-to-proliferative switch [61]. Interestingly, NFIs have been shown to be important modulators of TGFβ signaling [62], [63], providing an appealing molecular framework for the integration of NFI activity, hepatocyte differentiation and downstream effects on xenobiotic metabolism.

In summary, our study reveals that the NFI transcription factor family plays an important role in the control of hepatocyte differentiation and function. Specifically, NFIs can serve as a molecular link between demographic (age), physiological (inflammation) and environmental factors (alcohol intake) and CYP activity. These findings provide an additional puzzle piece of the molecular networks underlying CYP regulation in human liver with important implications for the understanding of interindividual differences in human drug metabolism.

## CRediT authorship contribution statement

**Kathrin Klein:** Writing – review & editing, Writing – original draft, Visualization, Supervision, Methodology, Formal analysis, Conceptualization. **Oliver Burk:** Writing – review & editing, Writing – original draft, Resources, Methodology, Investigation, Conceptualization. **Roman Tremmel:** Writing – review & editing, Writing – original draft, Software, Resources, Methodology, Formal analysis. **Florian A. Buettner:** Writing – original draft, Software, Methodology, Formal analysis, Data curation. **Lea Kaestle:** Methodology, Investigation, Formal analysis. **Werner Schroth:** Writing – original draft, Resources, Methodology, Investigation, Formal analysis, Data curation. **Thomas E. Muerdter:** Resources, Investigation, Formal analysis. **Diana M. Eccles:** Resources, Methodology. **Anna-Christina Schmidt:** Resources, Methodology. **Hanno Nieß:** Resources, Methodology. **Ulrich M. Zanger:** Resources, Methodology. **Matthias Schwab:** Writing – review & editing, Writing – original draft, Supervision, Resources, Project administration, Investigation, Funding acquisition, Formal analysis. **Volker M. Lauschke:** Writing – review & editing, Writing – original draft, Supervision, Investigation, Funding acquisition, Conceptualization.

## Ethics approvals

All studies used in this work complied with the Declaration of Helsinki. Written informed consent was obtained from all participants as required by institutional review boards and research ethics committees. All study samples and clinical information were completely anonymized prior use. The studies were approved by the Ethics Committees in Germany as follows: The use of human liver samples and clinical information was approved by the ethical committees of the University Medical Center Charité Berlin (# 88/99) and the University Hospital Tübingen (# 258/2009BO1, # 146/2012BO2). The TAM study was approved by the Medical Faculty of the University of Tübingen (# 893/2018BQ2 (formerly 340/2004)), and by the South West Multi-center Research Ethics Committee UK (# MREC 00/6/69). The SPART study was approved by the local ethical committee of the Robert Bosch Hospital Stuttgart (# 11–1991/06). The use of primary human hepatocytes isolated and provided by HTCR was approved by the ethics committee of the Faculty of Medicine at the Ludwig-Maximilians University München (# 025–12) as well as the Bavarian State Medical Association (# 11142) including regulation of informed written patient consent, data protection and anonymization.

## Declaration of Competing Interest

The authors declare the following financial interests/personal relationships which may be considered as potential competing interests: Oliver Burk reports equipment, drugs, or supplies was provided by HTCR foundation. Volker M. Lauschke reports a relationship with Ming Wai Lau Center for Reparative Medicine and the SciLifeLab and Wallenberg National Program for Data-Driven Life Science that includes: funding grants. Volker M. Lauschke reports a relationship with HepaPredict AB that includes: board membership, consulting or advisory fee, and equity or stocks ownership. Volker M. Lauschke reports a relationship with Shanghai Hepo Biotechnology Ltd that includes: board membership, consulting or advisory fee, and equity or stocks ownership. If there are other authors, they declare that they have no known competing financial interests or personal relationships that could have appeared to influence the work reported in this paper.

## Data Availability

Data will be made available on request.

## References

[1] Evans W.E., Relling M.V. (1999). Pharmacogenomics: translating functional genomics into rational therapeutics. Science.

[2] Meyer U.A. (2004). Pharmacogenetics - five decades of therapeutic lessons from genetic diversity. Nat. Rev. Genet.

[3] Jackson K.D., Achour B., Lee J., Geffert R.M., Beers J.L., Latham B.D. (2023). Novel approaches to characterize individual drug metabolism and advance precision Medicine. Drug Metab. Dispos..

[4] Klein K., Zanger U.M. (2013). Pharmacogenomics of cytochrome P450 3A4: recent progress toward the "missing heritability" problem. Front Genet.

[5] Ingelman-Sundberg M. (2022). The missing heritability in pharmacogenomics: role of NFIB and other factors. Pharmacogenomics.

[6] Harris L., Genovesi L.A., Gronostajski R.M., Wainwright B.J., Piper M. (2015). Nuclear factor one transcription factors: divergent functions in developmental versus adult stem cell populations. Dev. Dyn..

[7] Saito T., Takahashi Y., Hashimoto H., Kamataki T. (2001). Novel transcriptional regulation of the human CYP3A7 gene by Sp1 and Sp3 through nuclear factor kappa B-like element. J. Biol. Chem..

[8] Riffel A.K., Schuenemann E., Vyhlidal C.A. (2009). Regulation of the CYP3A4 and CYP3A7 promoters by members of the nuclear factor I transcription factor family. Mol. Pharm..

[9] Smith R.L., O'Connell K., Athanasiu L., Djurovic S., Kringen M.K., Andreassen O.A., Molden E. (2020). Identification of a novel polymorphism associated with reduced clozapine concentration in schizophrenia patients-a genome-wide association study adjusting for smoking habits. Transl. Psychiatry.

[10] Okhuijsen-Pfeifer C., van der Horst M.Z., Bousman C.A., Lin B., van Eijk K.R., Ripke S., Ayhan Y., Babaoglu M.O., Bak M., Alink W., van Beek H., Beld E., Bouhuis A., Edlinger M., Erdogan I.M., Ertuğrul A., Yoca G., Everall I.P., Görlitz T., Grootens K.P., Gutwinski S., Hallikainen T., Jeger-Land E., de Koning M., Lähteenvuo M., Legge S.E., Leucht S., Morgenroth C., Müderrisoğlu A., Narang A., Pantelis C., Pardiñas A.F., Oviedo-Salcedo T., Schneider-Thoma J., Schreiter S., Repo-Tiihonen E., Tuppurainen H., Veereschild M., Veerman S., de Vos M., Wagner E., Cohen D., Bogers J.P.A.M., Walters J.T.R., Yağcıoğlu A.E.A., Tiihonen J., Hasan A., Luykx J.J. (2022). Genome-wide association analyses of symptom severity among clozapine-treated patients with schizophrenia spectrum disorders. Transl. Psychiatry.

[11] Lenk H.Ç., Løvsletten Smith R., O'Connell K.S., Jukić M.M., Kringen M.K., Andreassen O.A., Ingelman-Sundberg M., Molden E. (2023). Impact of NFIB and CYP1A variants on clozapine serum concentration-A retrospective naturalistic cohort study on 526 patients with known smoking habits. Clin. Transl. Sci..

[12] Lenk H.Ç., Klöditz K., Johansson I., Smith R.L., Jukić M.M., Molden E., Ingelman-Sundberg M. (2022). The polymorphic nuclear factor NFIB regulates hepatic CYP2D6 expression and influences risperidone metabolism in psychiatric patients. Clin. Pharmacol. Ther..

[13] Smith R.L., Wollmann B.M., Størset E., Lenk H.Ç., O'Connell K.S., Kristiansen M.K., Ingelman-Sundberg M., Molden E. (2024). Effect of the NFIB rs28379954 TC polymorphism on CYP2D6-catalyzed metabolism of solanidine. Clin. Transl. Sci..

[14] Toscano C., Raimundo S., Klein K., Eichelbaum M., Schwab M., Zanger U.M. (2006). A silent mutation (2939GA, exon 6; CYP2D6*59) leading to impaired expression and function of CYP2D6. Pharm. Genom..

[15] Gomes A.M., Winter S., Klein K., Turpeinen M., Schaeffeler E., Schwab M., Zanger U.M. (2009). Pharmacogenomics of human liver cytochrome P450 oxidoreductase: multifactorial analysis and impact on microsomal drug oxidation. Pharmacogenomics.

[16] Klein K., Tremmel R., Winter S., Fehr S., Battke F., Scheurenbrand T., Schaeffeler E., Biskup S., Schwab M., Zanger U.M. (2019). A new Panel-Based Next-Generation sequencing method for ADME genes reveals novel associations of common and rare variants with expression in a human liver cohort. Front. Genet.

[17] Tremmel R., Nies A.T., van Eijck B.A.C., Handin N., Haag M., Winter S., Büttner F.A., Kölz C., Klein F., Mazzola P., Hofmann U., Klein K., Hoffmann P., Nöthen M.M., Gaugaz F.Z., Artursson P., Schwab M., Schaeffeler E. (2022). Hepatic expression of the Na+-Taurocholate cotransporting polypeptide is independent from genetic variation. Int. J. Mol. Sci..

[18] Weiß F., Hammer H.S., Klein K., Planatscher H., Zanger U.M., Norén A., Wegler C., Artursson P., Joos T.O., Poetz O. (2018). Direct quantification of cytochromes P450 and drug Transporters-A rapid, targeted mass Spectrometry-Based immunoassay panel for tissues and cell culture lysates. Drug Metab. Dispos..

[19] Khor C.C., Winter S., Sutiman N., Mürdter T.E., Chen S., Lim J.S.L., Li Z., Li J., Sim K.S., Ganchev B., Eccles D., Eccles B., Tapper W., Zgheib N.K., Tfayli A., Ng R.C.H., Yap Y.S., Lim E., Wong M., Wong N.S., Ang P.C.S., Dent R., Tremmel R., Klein K., Schaeffeler E., Zhou Y., Lauschke V.M., Eichelbaum M., Schwab M., Brauch H.B., Chowbay B., Schroth W. (2023). Cross-Ancestry Genome-Wide association study defines the extended CYP2D6 locus as the principal genetic determinant of endoxifen plasma concentrations. Clin. Pharmacol. Ther..

[20] Griese E.U., Zanger U.M., Brudermanns U., Gaedigk A., Mikus G., Mörike K., Stüven T., Eichelbaum M. (1998). Assessment of the predictive power of genotypes for the in-vivo catalytic function of CYP2D6 in a German population. Pharmacogenetics.

[21] Raimundo S., Toscano C., Klein K., Fischer J., Griese E.-U., Eichelbaum M., Schwab M. (2004). U.M. Zanger, a novel intronic mutation, 2988GA, with high predictivity for impaired function of cytochrome P450 2D6 in White subjects. Clin. Pharma Ther..

[22] Gaedigk A., Dinh J.C., Jeong H., Prasad B., Leeder J.S. (2018). Ten years' experience with the CYP2D6 activity score: a perspective on future investigations to improve clinical predictions for precision therapeutics. J. Pers. Med..

[23] Knobeloch D., Ehnert S., Schyschka L., Büchler P., Schoenberg M., Kleeff J., Thasler W.E., Nussler N.C., Godoy P., Hengstler J., Nussler A.K. (2012). Human hepatocytes: isolation, culture, and quality procedures. Methods Mol. Biol..

[24] Lee S.M.L., Schelcher C., Demmel M., Hauner M., Thasler W.E. (2013). Isolation of human hepatocytes by a two-step collagenase perfusion procedure. J. Vis. Exp..

[25] Thasler W.E., Weiss T.S., Schillhorn K., Stoll P.-T., Irrgang B., Jauch K.-W. (2003). Charitable State-Controlled foundation human tissue and cell research: ethic and legal aspects in the supply of surgically removed human tissue for research in the academic and commercial sector in Germany. Cell Tissue Bank.

[26] Burk O., Arnold K.A., Nussler A.K., Schaeffeler E., Efimova E., Avery B.A., Avery M.A., Fromm M.F., Eichelbaum M. (2005). Antimalarial artemisinin drugs induce cytochrome P450 and MDR1 expression by activation of xenosensors pregnane x receptor and constitutive androstane receptor. Mol. Pharm..

[27] Bitter A., Rümmele P., Klein K., Kandel B.A., Rieger J.K., Nüssler A.K., Zanger U.M., Trauner M., Schwab M., Burk O. (2015). Pregnane x receptor activation and silencing promote steatosis of human hepatic cells by distinct lipogenic mechanisms. Arch. Toxicol..

[28] Ewels P.A., Peltzer A., Fillinger S., Patel H., Alneberg J., Wilm A., Garcia M.U., Di Tommaso P., Nahnsen S. (2020). The nf-core framework for community-curated bioinformatics pipelines. Nat. Biotechnol..

[29] Patro R., Duggal G., Love M.I., Irizarry R.A., Kingsford C. (2017). Salmon provides fast and bias-aware quantification of transcript expression. Nat. Methods.

[30] Elizarraras J.M., Liao Y., Shi Z., Zhu Q., Pico A.R., Zhang B. (2024). WebGestalt 2024: faster gene set analysis and new support for metabolomics and multi-omics. Nucleic Acids Res.

[31] Feidt D.M., Klein K., Hofmann U., Riedmaier S., Knobeloch D., Thasler W.E., Weiss T.S., Schwab M., Zanger U.M. (2010). Profiling induction of cytochrome p450 enzyme activity by statins using a new liquid chromatography-tandem mass spectrometry cocktail assay in human hepatocytes. Drug Metab. Dispos..

[32] Kugler N., Klein K., Zanger U.M. (2020). MiR-155 and other microRNAs downregulate drug metabolizing cytochromes P450 in inflammation. Biochem Pharm..

[33] Tang D., Chen M., Huang X., Zhang G., Zeng L., Zhang G., Wu S., Wang Y. (2023). SRplot: a free online platform for data visualization and graphing. PLoS ONE.

[34] Brücker L., Jacob D., Preiss L.C., Zhong Y., Geist F., Hewitt P., Lauschke V.M., Petersson C. (2025). Evaluation of small interfering RNA-dependent knockdowns of drug-metabolizing enzymes in multiwell array culture of primary human hepatocyte spheroids for estimation of fraction metabolized. Drug Metab. Dispos..

[35] Olsavsky Goyak K.M., Laurenzana E.M., Omiecinski C.J. (2010). Hepatocyte differentiation. Methods Mol. Biol..

[36] Kim D.-S., Ryu J.-W., Son M.-Y., Oh J.-H., Chung K.-S., Lee S., Lee J.-J., Ahn J.-H., Min J.-S., Ahn J., Kang H.M., Kim J., Jung C.-R., Kim N.-S., Cho H.-S. (2017). A liver-specific gene expression panel predicts the differentiation status of in vitro hepatocyte models. Hepatology.

[37] Gao X., Li R., Cahan P., Zhao Y., Yourick J.J., Sprando R.L. (2020). Hepatocyte-like cells derived from human induced pluripotent stem cells using small molecules: implications of a transcriptomic study. Stem Cell Res. Ther..

[38] Ueyama-Toba Y., Tong Y., Yokota J., Murai K., Hikita H., Eguchi H., Takehara T., Mizuguchi H. (2024). Development of a hepatic differentiation method in 2D culture from primary human hepatocyte-derived organoids for pharmaceutical research. iScience.

[39] Lauschke V.M., Ingelman-Sundberg M. (2019). Prediction of drug response and adverse drug reactions: from twin studies to next generation sequencing. Eur. J. Pharm. Sci..

[40] Matthaei J., Brockmöller J., Tzvetkov M.V., Sehrt D., Sachse-Seeboth C., Hjelmborg J.B., Möller S., Halekoh U., Hofmann U., Schwab M., Kerb R. (2015). Heritability of metoprolol and torsemide pharmacokinetics. Clin. Pharmacol. Ther..

[41] Zhou Y., Tremmel R., Schaeffeler E., Schwab M., Lauschke V.M. (2022). Challenges and opportunities associated with rare-variant pharmacogenomics. Trends Pharmacol. Sci..

[42] Lauschke V.M., Zhou Y., Ingelman-Sundberg M. (2024). Pharmacogenomics beyond single common genetic variants: the way forward. Annu. Rev. Pharmacol. Toxicol..

[43] Böhm R., Bruckmueller H., Oswald S., Hübenthal M., Kaehler M., Ehmke L., Höcker J., Siegmund W., Franke A., Cascorbi I. (2024). Phenotype-Genotype correlation applying a cocktail approach and an exome chip analysis reveals further variants contributing to variation of drug metabolism. Clin. Pharmacol. Ther..

[44] Narvaez M.J., Anderson G.R., Pickwell G.V., Quattrochi L.C. (2005). Characterization of adjacent E-box and nuclear factor 1-like DNA binding sequence in the human CYP1A2 promoter. J. Biochem. Mol. Toxicol..

[45] Zhang J., Zhang Q.Y., Guo J., Zhou Y., Ding X. (2000). Identification and functional characterization of a conserved, nuclear factor 1-like element in the proximal promoter region of CYP1A2 gene specifically expressed in the liver and olfactory mucosa. J. Biol. Chem..

[46] Pinar D.M., Göös H., Tan Z., Kumpula E.-P., Chowdhury I., Wang Z., Zhang Q., Salokas K., Keskitalo S., Wei G.-H., Kumbasar A., Varjosalo M. (2024). Nuclear factor I family members are key transcription factors regulating gene expression. Mol. Cell. Proteom..

[47] Fraser J., Essebier A., Brown A.S., Davila R.A., Harkins D., Zalucki O., Shapiro L.P., Penzes P., Wainwright B.J., Scott M.P., Gronostajski R.M., Bodén M., Piper M., Harvey T.J. (2020). Common regulatory targets of NFIA, NFIX and NFIB during postnatal cerebellar development. Cerebellum.

[48] Göös H., Kinnunen M., Salokas K., Tan Z., Liu X., Yadav L., Zhang Q., Wei G.-H., Varjosalo M. (2022). Human transcription factor protein interaction networks. Nat. Commun..

[49] Lamani E., Wu Y., Dong J., Litaker M.S., Acevedo A.C., MacDougall M. (2009). Tissue- and cell-specific alternative splicing of NFIC. Cells Tissues Organs.

[50] Singh S.K., Wilczynska K.M., Grzybowski A., Yester J., Osrah B., Bryan L., Wright S., Griswold-Prenner I., Kordula T. (2011). The unique transcriptional activation domain of nuclear factor-I-X3 is critical to specifically induce marker gene expression in astrocytes. J. Biol. Chem..

[51] Messina G., Biressi S., Monteverde S., Magli A., Cassano M., Perani L., Roncaglia E., Tagliafico E., Starnes L., Campbell C.E., Grossi M., Goldhamer D.J., Gronostajski R.M., Cossu G. (2010). Nfix regulates fetal-specific transcription in developing skeletal muscle. Cell.

[52] Piper M., Barry G., Hawkins J., Mason S., Lindwall C., Little E., Sarkar A., Smith A.G., Moldrich R.X., Boyle G.M., Tole S., Gronostajski R.M., Bailey T.L., Richards L.J. (2010). NFIA controls telencephalic progenitor cell differentiation through repression of the notch effector Hes1. J. Neurosci..

[53] Heng Y.H.E., McLeay R.C., Harvey T.J., Smith A.G., Barry G., Cato K., Plachez C., Little E., Mason S., Dixon C., Gronostajski R.M., Bailey T.L., Richards L.J., Piper M. (2014). NFIX regulates neural progenitor cell differentiation during hippocampal morphogenesis. Cereb. Cortex.

[54] Piper M., Barry G., Harvey T.J., McLeay R., Smith A.G., Harris L., Mason S., Stringer B.W., Day B.W., Wray N.R., Gronostajski R.M., Bailey T.L., Boyd A.W., Richards L.J. (2014). NFIB-mediated repression of the epigenetic factor Ezh2 regulates cortical development. J. Neurosci..

[55] Chang C.-Y., Pasolli H.A., Giannopoulou E.G., Guasch G., Gronostajski R.M., Elemento O., Fuchs E. (2013). NFIB is a governor of epithelial-melanocyte stem cell behaviour in a shared niche. Nature.

[56] Martynoga B., Mateo J.L., Zhou B., Andersen J., Achimastou A., Urbán N., van den Berg D., Georgopoulou D., Hadjur S., Wittbrodt J., Ettwiller L., Piper M., Gronostajski R.M., Guillemot F. (2013). Epigenomic enhancer annotation reveals a key role for NFIX in neural stem cell quiescence. Genes Dev..

[57] Holmfeldt P., Pardieck J., Saulsberry A.C., Nandakumar S.K., Finkelstein D., Gray J.T., Persons D.A., McKinney-Freeman S. (2013). Nfix is a novel regulator of murine hematopoietic stem and progenitor cell survival. Blood.

[58] Heinke P., Rost F., Rode J., Trus P., Simonova I., Lázár E., Feddema J., Welsch T., Alkass K., Salehpour M., Zimmermann A., Seehofer D., Possnert G., Damm G., Druid H., Brusch L., Bergmann O. (2022). Diploid hepatocytes drive physiological liver renewal in adult humans. Cell Syst..

[59] Michalopoulos G.K., Bhushan B. (2021). Liver regeneration: biological and pathological mechanisms and implications. Nat. Rev. Gastroenterol. Hepatol..

[60] Wang M.-J., Chen F., Li J.-X., Liu C.-C., Zhang H.-B., Xia Y., Yu B., You P., Xiang D., Lu L., Yao H., Borjigin U., Yang G.-S., Wangensteen K.J., He Z.-Y., Wang X., Hu Y.-P. (2014). Reversal of hepatocyte senescence after continuous in vivo cell proliferation. Hepatology.

[61] Oliva-Vilarnau N., Beusch C.M., Sabatier P., Sakaraki E., Tjaden A., Graetz L., Büttner F.A., Dorotea D., Nguyen M., Bergqvist F., Sundström Y., Müller S., Zubarev R.A., Schulte G., Tredup C., Gramignoli R., Tietge U.J.F., Lauschke V.M. (2024). Wnt/β-catenin and NFκB signaling synergize to trigger growth factor-free regeneration of adult primary human hepatocytes. Hepatology.

[62] Rossi P., Karsenty G., Roberts A.B., Roche N.S., Sporn M.B., de Crombrugghe B. (1988). A nuclear factor 1 binding site mediates the transcriptional activation of a type I collagen promoter by transforming growth factor-beta. Cell.

[63] Plasari G., Calabrese A., Dusserre Y., Gronostajski R.M., McNair A., Michalik L., Mermod N. (2009). Nuclear factor I-C links platelet-derived growth factor and transforming growth factor beta1 signaling to skin wound healing progression. Mol. Cell. Biol..

