# Supplementary material for: Transcription factors of the Nuclear Factor I (NFI) family control hepatocyte differentiation and cytochrome P450 activity in human liver
Source: Pharmacol Res. 2025 Nov;221:107998. doi: 10.1016/j.phrs.2025.107998 (PMC12580495; doi:10.1016/j.phrs.2025.107998)
Supplement: Supplementary file 1 — Supplementary material [file mmc1.docx]

**SUPPLEMENTARY MATERIAL**

**Transcription factors of the nuclear factor I (NFI) family control hepatocyte differentiation and cytochrome P450 activity in human liver**

Kathrin Klein, Oliver Burk, Roman Tremmel, Florian A. Buettner, Lea Kaestle, Werner Schroth. Thomas E. Muerdter, Diana Eccles, Anna-Christina Schmidt, Hanno Nieß, Ulrich M. Zanger, Matthias Schwab, Volker M. Lauschke

**Supplementary Figures: 3**

**Supplementary Table: 4**

**Supplementary Methods**

**Supplementary References**

**Supplementary Figures**

**Figure S1: Representative Western blots for quantification of CYP isoforms in human liver microsomal samples (HLM).**

For each of the displayed CYP isoforms, one representative immunostained Western blot is shown. **CYP1A1**, n=15 HLM, 50µg/lane; **CYP1A2**, n=17 HLM, 10µg/lane; **CYP2A6,** n=17 HLM, 25µg/lane; **CYP2C8**, n=13 HLM, 10µg/lane; **CYP2C19**, n=15 HLM, 50µg/lane. Lanes with standard protein are marked and concentrations are indicated (fmols/lane), lanes with pooled microsomal reference sample (P) are marked. All lanes without label are HLM from human liver cohort. (see also: Supplementary Methods, page 11)

**
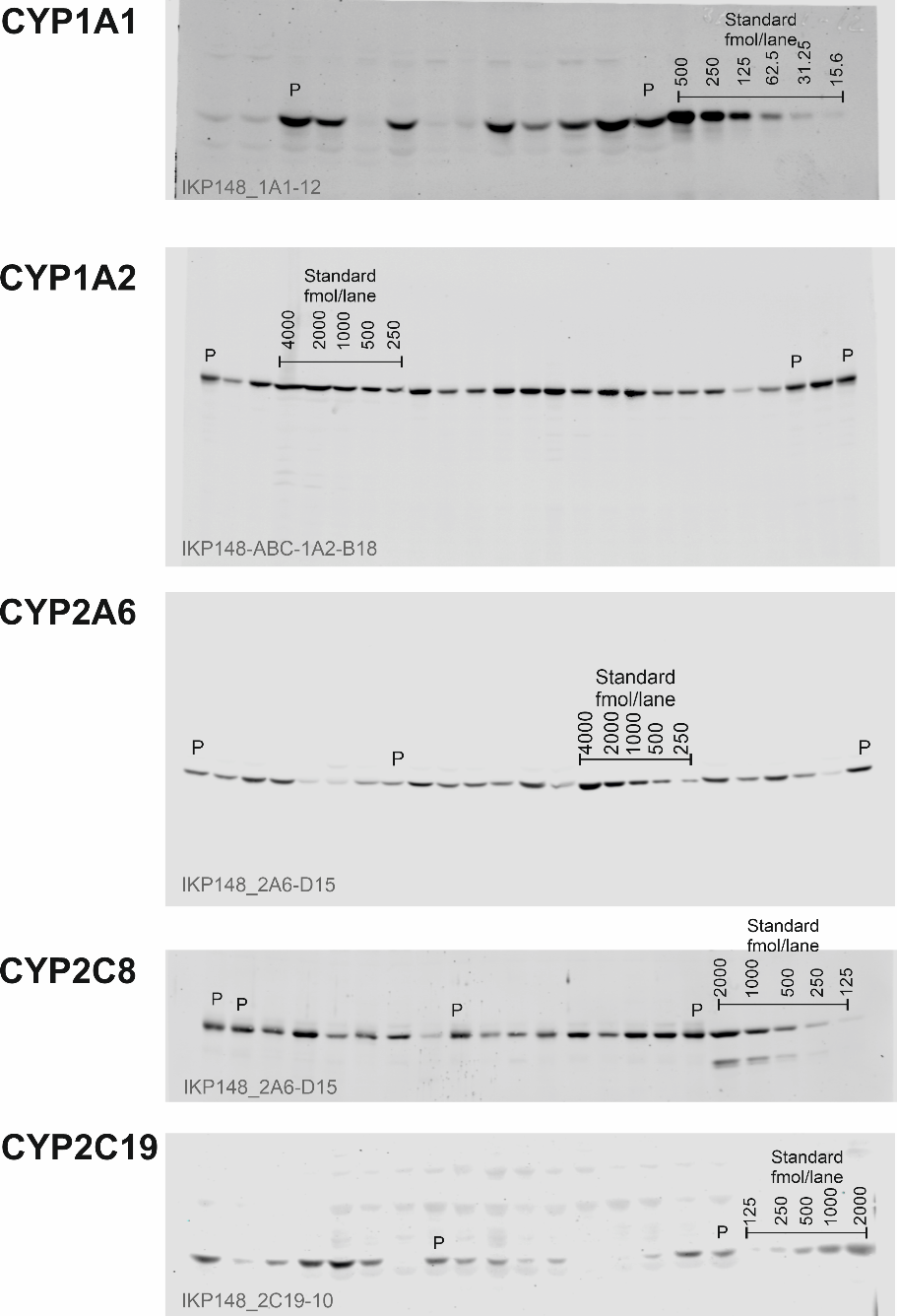
**

**Figure S2: Influence of non-genetic factors on *Nuclear Factor I* gene expression in human liver**

**A:** Correlation of NFIA (left) and NFIX (right) expression with age**.** x-axis: age given in years; y-axis expression (log2 transformed). **B-F:** NFI gene expression (log2 transformed) displayed as violin plots and values grouped by different non-genetic factors. Red line: median; quartiles in black. Only significant non-genetic factors are shown (see also Figure 2). **B:** alcohol consumption (ALC): no alcohol (n=77); occasional (n=32), weekly (n=6); **C:** cholestasis (CHOL), yes (n=21), no (n=93); **D:** C‑reactive protein (CRP), elevated levels (n=6), not elevated (n=109); **E:** underlying liver disease (DIAG): patients with isolated liver metastasis (n=76), patients with benign or malign primary liver tumors (n=41); **F:** co-medication (DRUG): no (n=31), yes (n=32). Wilcoxon *p*-values given (*, p < 0.05; **, p < 0.01; significance levels corresponding to Figure 2).

**
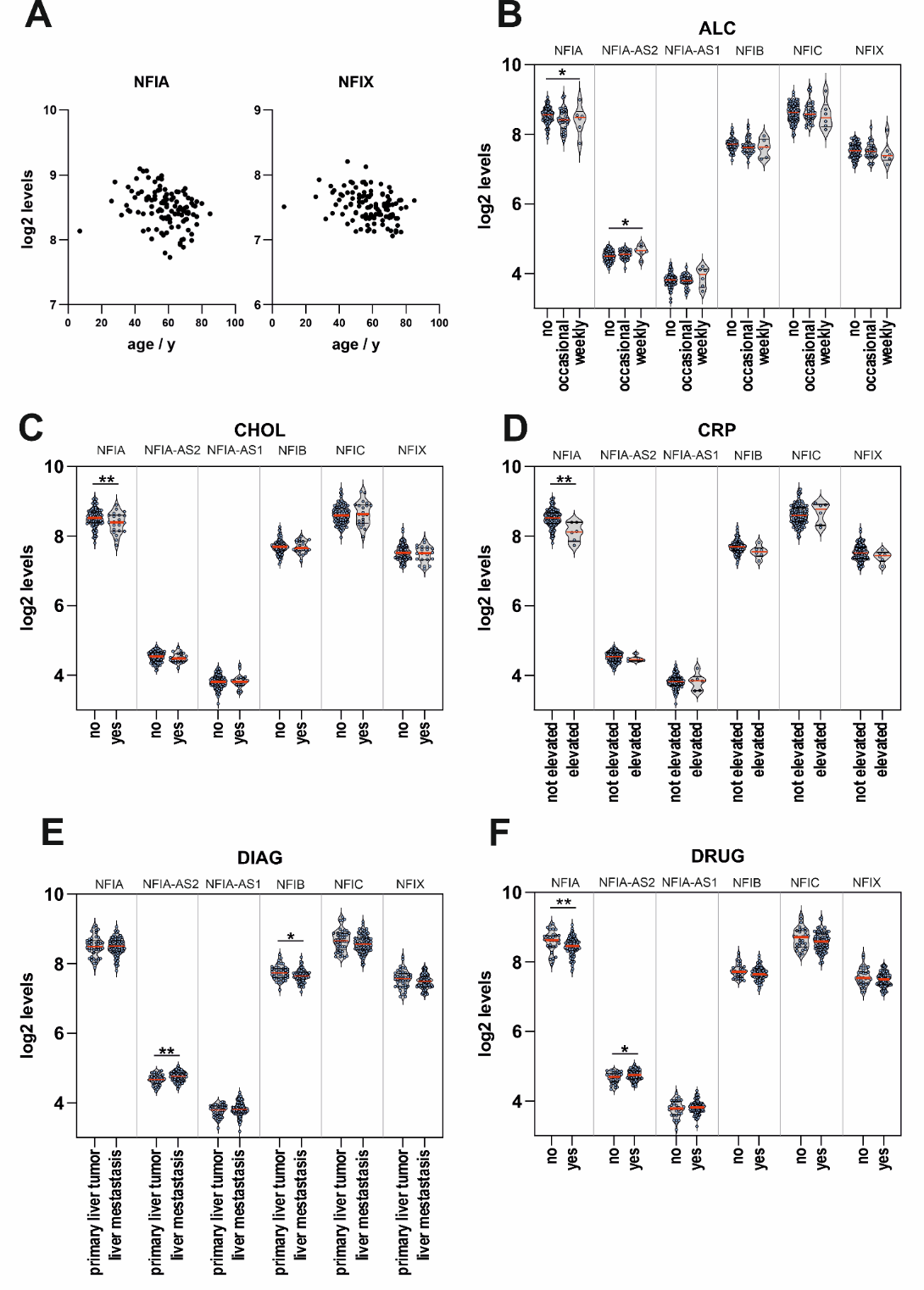
**

**Figure S3:** **Gene knockdown for the four coding NFI genes in primary human hepatocytes and effects on expression of selected CYP450 genes**.

**A:** relative *NFI* gene expression levels compared to non-targeting siRNA (siCTR) treatment, quantified for each NFI gene using realtime PCR analysis (TaqMan), showing the specificity for each siRNA treatment (n=5 donors). **B:** Effect of knockdown of the four NFI genes on the gene expression of seven selected CYP450 isozymes measured by realtime PCR (TaqMan) relative to non-targeting siRNA in hepatocyte donors (n=4). Individual relative values are given. Boxes illustrating the mean, whiskers showing the standard deviation. Statistical difference between siRNA treated and siCTRL treated cells was calculated by one sample t-test, and p-values are given: p<0.05(*); p<0.01(**); p<0.001(***).

**Supplementary Tables**

**Supplementary Table S1:** Liver cohort clinical documentation

Subgroup Number

**All 150**

**Sex**

Females 79

Males 71

**Age**

<20 2

≥20 and ≤70 122

>70 26

**Smoking status**

Non-smoker 117

3-20 cigarettes/day 18

>20 cigarettes/day 11

**Alcohol consumption**

None 96

1-2 times/week 38

Daily 10

**Presurgery medication**

No 40

Yes 110

**Supplementary Table S2:** NFIB_rs28379954_T>C genotype distribution in study cohorts

| **cohort** | **reference** | **Total  n** | **Total alleles** | **TT** | **TC** | **CC** | **Freq [C]^a^** | **Chi² ^b^** |
| --- | --- | --- | --- | --- | --- | --- | --- | --- |
| **TAM** | [1,2] | 579 | 1158 | 525 | 54 | 0 | 0.047 | 0.074 |
| **SPART** | [3,4] | 199 | 398 | 186 | 13 | 0 | 0.033 | 0.2281 |
| **Liver bank** | [5,6,7] | 150 | 300 | 138 | 11 | 1 | 0.043 | 2.00 |

^a^ frequency of variant [C] allele

^b^ Chi^2^ test (https://wpcalc.com/en/equilibrium-hardy-weinberg/)

**Supplementary Table S3:** Primary hepatocyte donor information

| **ID** | **ethnicity** | **sex** | **age** | **diagnosis** | **long-term medication** |
| --- | --- | --- | --- | --- | --- |
| GH68 | n.s. | Male | 64 | liver metastasis CRC | candesartan, insulin, simvastatin, apixaban, empagliflozin |
| GH69 | n.s. | Male | 58 | intrahepatic CCC | tamsulosin, atorvastatin, amlodipine, ramipril, metoprolol |
| GH70 | n.s. | Male | 84 | intrahepatic CCC | acetylsalicylic acid, atorvastatin, candesartan, finasteride, pantoprazole |
| GH71 | n.s. | Female | 78 | liver metastasis CRC | bisoprolol, hydrochloro-thiazide, metformin, sitagliptin |
| RFM | Caucasian | Female | 42 | drug intoxication | BioIVT, F00995-P |

n.s., not specified; CRC, colorectal carcinoma; CCC, cholangiocellular carcinoma

**Supplementary Table S4:** Gene expression assays

| **gene** | **type** | **assay ID^a^** |
| --- | --- | --- |
| NFIA | gene expression | Hs00325656_m1 |
| NFIB | gene expression | Hs01029174_m1 |
| NFIC | gene expression | Hs00232157_m1 |
| NFIX | gene expression | Hs00958846_m1 |
| ABCB11 | gene expression | Hs00184824_m1 |
| AFP | gene expression | Hs00173490_m1 |
| ALB | gene expression | Hs00910225_m1 |
| ANXA1 | gene expression | Hs00167549_m1 |
| APOB | gene expression | Hs01071209_m1 |
| APOE | gene expression | Hs00171168_m1 |
| CYP1A2 | gene expression | Hs00167927_m1 |
| CYP2C19 | gene expression | Hs00426380_m1 |
| CYP2C8 | gene expression | Hs00946140_g1 |
| CYP2C9 | gene expression | Hs00426397_m1 |
| CYP2D6 | gene expression | Hs00164385_m1 |
| CYP2E1 | gene expression | Hs00559368_m1 |
| CYP3A4 | gene expression | Hs00604506_m1 |
| CYP8B1 | gene expression | Hs00244754_s1 |
| FGA | gene expression | Hs00241027_m1 |
| GSTP1 | gene expression | Hs00168310_m1 |
| HNF1A | gene expression | Hs00167041_m1 |
| HNF4A | gene expression | Hs01023298_m1 |
| NR1I2 | gene expression | Hs00243666_m1 |
| NR1I3 | gene expression | Hs00901571_m1 |
| PON1 | gene expression | Hs00166557_m1 |
| SERPINC1 | gene expression | Hs00166654_m1 |
| TF | gene expression | Hs01067777_m1 |
| TTR | gene expression | Hs00174914_m1 |
| UGT2B4 | gene expression | Hs02383831_s1 |
| UGT2B7 | gene expression | Hs00426592_m1 |
| TBP | gene expression | Hs00427620_m1 |
| YWAHZ | gene expression | Hs01122445_g1 |

^a^ Life Technologies by Thermo Fisher

**Supplementary Methods**

**Western Blot series for 150 human liver microsomal samples (HLM).**

Each PAGE (10%) was run with a dilution row of standard protein (approximately 4,000–15 fmol) for the target CYP isoform, as well as a colored size marker. 10–50 µg of HLM samples were loaded alongside a pooled microsomal reference sample in two to four lanes. Semi-dry electro-blotting was performed with conditions optimized for each target protein. Each blot was stained with Ponceau solution to control for homogenous protein transfer on the nitrocellulose membrane. Immunostaining conditions with primary and secondary IRDye680LT- or IRDye800CW-labelled antibodies (Licor Biosciences) were performed according to the manufacturers' recommendations and adjusted in pre-tests for each target protein, if needed. The blots were documented using an Odyssey Imager (Licor Biosciences) The total set of 150 samples was distributed over at least 20–30 blots for one target protein and the quantification was recalculated using the standard row (Odyssey documentation software or Image Studio Lite). As control, a pooled HLM sample (P) was included on every blot for consistency of quantification throughout one blot and the complete series. Care was taken to ensure that lot numbers of standard proteins and antibodies did not change within a series. All blots and evaluations were visited by two people. Double or triple values were obtained for the samples and expressed in pmol CYP isozyme per mg microsomal protein. ***CYP1A1****, standard protein: human CYP1A1+OR Microsomes (BDGentest455111), mouse anti human CYP1A1 (clone1-599-16), IRDye800 (LICORbio 926-32210);* ***CYP1A2****, standard protein: human CYP1A2 Microsomes (BDGentest455103), mouse anti human CYP1A2 (clone26-7-5), anti mouse IRDye800 (LICORbio 926-32210);* ***CYP2A6,*** *standard protein: human CYP2A6+OR Microsomes (BDGentest455104), mouse anti human CYP2A6 (BDGentest 458106), anti mouse IRDye800 (LICORbio 926-32210); CYP2C8, standard protein: human CYP2C8+OR Microsomes (BDGentest455112), rabbit anti human CYP2C8 (Puracyp Hu-A004), anti rabbit IRDye680LT (LICORbio 926-68021);* ***CYP2C19****, standard protein: human CYP2C19 Microsomes (BDGentest455119), rabbit anti human CYP2C18 (BDGentest458219), anti rabbit IRDye680LT (LICORbio 926-68021).*  Protein data for the here used liver biobank were published [4-5]., Representative blots are shown in **Supplementary Figure S1** for each of the proteins that are significantly associated with NFI gene expression in 150 livers (see **Figure 3D**).

**References**

[1] Khor et al., (2023) C ross-Ancestry Genome-Wide Association Study Defines the Extended CYP2D6 Locus as the Principal Genetic Determinant of Endoxifen Plasma Concentrations, Clin. Pharmacol. Ther. 113:712–723. <https://doi.org/10.1002/cpt.2846>.

[2] Copson, et al., (2013) Prospective observational study of breast cancer treatment outcomes for UK women aged 18-40 years at diagnosis: the POSH study, J. Natl. Cancer Inst. 105:978–988. <https://doi.org/10.1093/jnci/djt134>

[3] Griese et al., (1998) Assessment of the predictive power of genotypes for the in-vivo catalytic function of CYP2D6 in a German population, Pharmacogenetics 8:15–26.
<https://doi.org/10.1097/00008571-199802000-00003>.

[4] Raimundo et al., (2004) A novel intronic mutation, 2988GA, with high predictivity for impaired function of cytochrome P450 2D6 in white subjects. Clin. Pharmacol. Ther. 76:128–138. <https://doi.org/10.1016/j.clpt.2004.04.009>.

[5] Toscano et al., (2006) A silent mutation (2939G>A, exon 6; CYP2D6*59) leading to impaired expression and function of CYP2D6. Pharmacogenet. Genomics 16:767-770.
<https://doi.org/10.1097/01.fpc.0000236331.03681.24>.

[6] Gomes et al., (2009) Pharmacogenomics of human liver cytochrome P450 oxidoreductase: multifactorial analysis and impact on microsomal drug oxidation, Pharmacogenomics 10:579–599. <https://doi.org/10.2217/pgs.09.7>.

[7] Tremmel et al., (2022) Hepatic Expression of the Na+-Taurocholate Cotransporting Polypeptide Is Independent from Genetic Variation, Int. J. Mol. Sci. 23:7468.
<https://doi.org/10.3390/ijms23137468>
